# Prostate Infiltration by Treg and Th17 Cells as an Immune Response to *Propionibacterium acnes* Infection in the Course of Benign Prostatic Hyperplasia and Prostate Cancer

**DOI:** 10.3390/ijms23168849

**Published:** 2022-08-09

**Authors:** Sebastian Radej, Monika Szewc, Ryszard Maciejewski

**Affiliations:** 1 Department of Normal Anatomy, Medical University of Lublin, 20-090 Lublin, Poland; 2 Institute of Health Sciences, The John Paul II Catholic University of Lublin, 20-708 Lublin, Poland

**Keywords:** prostate cancer, benign prostatic hyperplasia, prostate microbiome, *P. acnes*, inflammatory cells, Treg cells, Th17 cells, prostate chronic inflammation

## Abstract

Benign prostatic hyperplasia (BPH) and prostate cancer (PCa) belong to the most frequent diseases in ageing men. It has been proposed that prostate chronic inflammation is a risk factor for the development of both BPH and PCa. However, potential stimuli that cause or maintain inflammation in the prostate gland are still poorly characterized. Bacterial infections seems to be one of the potential sources of prostatitis. Recent studies show that *Propionibacterium acnes (P. acnes)* is the most prevalent microorganism in the prostate gland and may be a predisposing factor for inflammation of prostatic tissue. It indicates that *P. acnes* may contribute to cancer development by enhancing proinflammatory responses, as well as by modifying the prostate extracellular environment. In this review, we discuss the potential role of *P. acnes* in the development of BPH and PCa and highlight the importance of regulatory T CD4(+)FoxP3(+) (Treg) and Th17 cells in response to *P. acnes* infection in the context of both prostate diseases.

## 1. Introduction

Benign prostatic hyperplasia (BPH) and prostate cancer (PCa) belong to the most frequent diseases in ageing men [1,2,3]. BPH is a nonmalignant enlargement of the prostate gland caused by unregulated hypertrophy of the epithelial and fibromuscular tissues of the transition zone (TZ) and periurethral area. It is a common cause of lower urinary tract symptoms (LUTS) in men. Patients with BPH may experience poor urinary flow, frequency, hesitancy initiating flow, post-void dribbling, and nocturia. The prevalence of the disease increases after the age of 40 years [2,3,4]. So far, emerging hypotheses to explain the pathogenesis of BPH have included androgen, estrogen, insulin, stem cell, proliferative reawakening, telomerase, and inflammatory pathways. Currently, the leading area of discussion and research on the etiology of this disease is chronic inflammation within the prostate gland, which causes growth factor production, stem cell activation, and cellular proliferation [4]. Potential stimuli for the inflammatory process and, consequently for the BPH, have been proposed. These include autoimmune responses, bacterial and viral infections, dietary factors, hormone changes, and urinary reflux into the collecting ducts of the prostate [5]. The mentioned stimuli may cause lymphocyte activation, cytokine release, and growth factor, which induce hyperplasia acts as a self-perpetuating cycle, leading to chronic inflammation and a progressive increase in prostate volume [6]. 

Prostate cancer (PCa) is the first most common malignancy in men and the second leading cause of cancer death among men worldwide (2021 estimate) [7]. It develops in elderly men and is rare in men under 40. The average age at diagnosis is about 66 [1]. In addition to ageing, well-established risk factors for PCa include family history of disease, certain inherited genetic conditions (e.g., Lynch syndrome and *BRCA1* and *BRCA2* mutations) and African ancestry [8]. There is also evidence that smoking and excess body weight may increase the risk of fatal PCa [9,10]. Early-stage disease is asymptomatic. In more advanced cases, PCa symptoms are similar to benign prostate conditions, such as BPH and prostatitis [1]. The high long-term survival is observed in patients with localized prostate cancer. However, metastatic prostate cancer remains largely incurable even despite the use of intensive multimodal therapy. The mortality of advanced disease is due to the lack of therapeutic regiments capable of generating durable responses in conditions of extreme tumor heterogeneity at the genetic and biological cell levels [11].

The three main causes of prostate-related morbidity are BPH, PCa, and prostatitis. Despite many years of scientific study, the etiology and pathogenesis of BPH and PCa have not been fully understood. Currently, researchers have focused their attention on the role of prostate chronic inflammation in the development of both diseases [12,13]. The frequent observation of inflammatory cells in the prostate microenvironment in adult men indicates that inflammation is involved in these conditions [14]. Recent evidence points to a role for inflammation and atrophy in the development of prostate diseases, and suggests that the prostate microbiome may be involved in establishing an inflammatory microenvironment of the prostate that may promote carcinogenesis and tumor progression [15,16]. Bacterial infections are one of the potential stimuli that cause or maintain tissue inflammation [17,18]. As research shows, *Propionibacterium ances* (*P. acnes*) is the most prevalent microorganism isolated from prostatic tissue. There are studies suggesting that *P. acnes* contributes to the development of prostate inflammation, and consequently, to prostate diseases. This bacterium is involved in the inflammatory response by producing chemotactic factors and attracting leucocytes. *P. acnes* arouses a particular interest in the discussed context. Therefore, in this review, we discuss the potential role of *P. acnes* in the development of BPH and PCa and highlight the importance of regulatory T CD4(+)FoxP3(+) (Treg) and Th17 cells in response to *P. acnes* infection in the context of both prostate diseases.

## 2. The Prostate Microbiome and Chronic Inflammation

Chronic inflammation is commonly observed in patients with BPH, preneoplastic and malignant prostates. Hence, it has been suggested that chronic inflammation is the risk factor for the development of BPH, prostate carcinogenesis and cancer progression. More than 150 years ago, the link between inflammation and cancer was hypothesized by Virchow after his discovery of leukocytes in neoplastic tissues. Research has shown that inflammation may contribute to the development of cancer in many organs, for instance, in the bladder, colon, liver, lungs, pancreas, and prostate [19]. The molecular evidence presented so far, from animal and human studies, points to the regulatory role of chronic inflammation in prostate cancer development and progression to advanced metastatic disease [19,20,21,22]. The presence of chronic prostatitis was identified to be an independent marker for Gleason score upgrade (GSU) by Guner et al. The researchers compared two groups with and without GSU in terms of chronic prostatitis. The study showed that the presence of chronic prostatitis associated with PCa was higher in the patient cohort with GSU in contrast to the other group [23].

According to the researchers, there are some potential stimuli of prostatic inflammation: microbial infections, chemical irritations, diet, obesity, and physical traumas [24,25]. These factors can cause DNA damage and epithelial injury. The epithelial damage triggers an immune system response leading to the expansion and recruitment of the inflammatory cells to the prostate. These cells produce cytokines, chemokines, and free radicals which cause chronic inflammation, DNA damage, and further epithelial injury. In consequence, it leads to compensatory epithelial proliferation and the nuclear alternations. Thus, prostatic intraepithelial neoplasia may occur [24].

**Figure 1:**
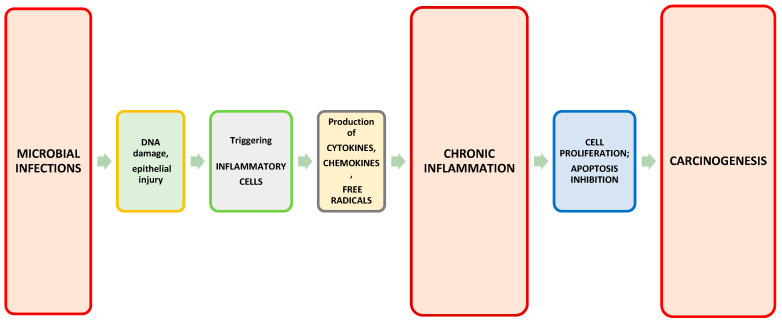
Microbial infections as a cause of chronic inflammation that can lead to carcinogenesis.

The effect of the prostate microbiome on prostate diseases is of particular interest. The microbiome can influence every stage of the disease from initiation to progression and treatment outcomes. This may occur as a result of direct interactions with a known microbial etiology, as well as modulation of the immune system, changes in metabolism, and effects on therapy. In many cases, both direct and indirect interactions with the microbiome are involved [26]. As many studies show, microbial inflammation is associated with the stimulation of the production of cytokines and chemokines. This can lead to cell proliferation and/or inhibition of apoptosis. Subsequently, carcinogenesis may be promoted (Figure 1) [27,28]. In healthy individuals, it has been observed that differentiated bacterial flora causes the production of inflammatory cytokines, including IFNγ, TNF-α, IL-1β, IL-6, IL-17, and IL-22 by myeloid and lymphoid cells [29]. Tumor progression can be promoted by these factors through various mechanisms, including IFNγ and IL-17 mediated tumor immune surveillance or the recruitment of immune cells into the tumor microenvironment via TNF-α, IL-1β, and IL-6 [28].

The prostate gland can be chronically exposed to a multitude of microorganisms. It indicates that there are associations between the microbiome composition and pathological conditions of prostatic tissue. Epidemiological, histopathological, and molecular data suggest that prostate chronic inflammation is connected with bacterial and viral infections. Research shows that as much as 10–20% of cancers are attributed to chronic inflammation involving microbes [22]. As studies show, DNA and RNA from bacteria, fungi, parasites, and viruses have been found in prostatectomy samples from men who suffer from BPH and PCa [30,31,32]. Specific microbes can cause genome instability and, consequently, influence carcinogenesis by producing tumor-promoting metabolites and inducing an immune response [16]. Therefore, studies have indicated that the prostate tissue microbiome can contribute to prostate inflammation in relation to benign prostate conditions such as BPH, as well as to tumor progression and the response to treatment [26,33,34].

As research shows, the prostatic tissue contains a variety of bacteria. The microbiome of the prostate tumor microenvironment was analyzed by Cavarretta et al. In this study, the authors noted that *Propionibacterium* spp. were the most abundant among other genera and that *Staphylococcus* spp. were more represented in the tumor tissues [35]. In other research, Feng et al. assessed the metagenome and metatranscriptome of prostatic tissue and they found that *Propionibacterium, Escherichia, Acinetobacter, and Pseudomonas* spp. were abundant and constituting the core of the prostate microbiome both in tumor and in adjacent benign tissues [36]. Yow et al. used 16S rRNA gene sequencing to detect bacterial agents in high-grade prostate cancer tissues. The authors identified *Enterobacteriaceae* spp. common to all examined samples and *P. acnes* in 95% of analyzed samples [37]. The presence of *P. acnes.* in the prostate gland appears to be crucial, as evidenced by numerous studies examining its potential role in the development of prostate diseases.

## 3. *P. acnes* in Patients with BPH and PCa

*P. acnes* has been reported as the most prevalent microorganism in normal and pathological prostate glands [32,35]. It is an anaerobic, Gram-positive, and opportunistic bacterium whose occurrence on the skin is the common cause of *acne vulgaris* [38]. However, as research shows, prostate-derived *P. acnes* isolates do not represent contamination from patient skin, the medical team, or the surgical environment. The researchers typed *P. acnes* isolates from radical prostatectomy tissue samples using multilocus sequence typing (MLST). They identified eight different sequence types (STs) among prostate-derived *P. acnes* isolates. Interestingly, these were not typical skin/acne STs, but rather characteristic STs associated with opportunistic infections and/or urethral flora [39]. It appears that urinary microbial studies are important in identifying prostate diseases [16,26,40,41]. 

As a ubiquitous slow-growing organism with the capacity to form biofilm, *P. acnes* has been also identified as the etiological agent in implant-associated infections, related to, for example, prosthetic heart valves, prosthetic joint devices, and neurosurgical shunts. However, the virulence of *P. acnes* is low. Therefore, infection symptoms occur after a long-term infection [42,43]. *P. acnes* may also be involved in sarcoidosis pathogenesis [44,45].

Recent research shows that *P. acnes* is isolated with a high frequency from prostatic tissue of patients with BPH and PCa [32,35,46]. Cavaretta et al. noticed the high abundance of *Propionibacterium* spp., mainly composed by *P. acnes*, in non-tumor and tumor prostatic tissue [35]. The study carried out by Davidsson et al. showed that *P. acnes* is more common in men with PCa (60% of cases with *P. acnes*) than in men without neoplastic lesions in the prostate gland (only 26% cases with *P.acnes*) [46]. Other researchers, using in situ immunofluorescence (ISIF), found *P. acnes* in 58 out of 71 (82%) tested cancerous prostate tissue samples. However, in the same study, *P. acnes* was absent in healthy prostate tissues (20 samples) [47]. Dadashi et al. detected *P. acnes* in 68% of PCa and 58% of BPH specimens [48]. *P. acnes* has also been shown by Alexeyev O. et al. as the predominant microorganism in prostatic tissue in a large cohort of BPH patients [32]. Other authors also confirmed the high frequency of *P. acnes* isolation from individuals with BPH (positive *P. acnes* in 41% of cases) [49]. Cohen et al. observed a significant higher degree of prostatic inflammation in prostate samples positive for *P. acnes* [50]. It has also been reported that the *P. acnes* detection in prostate tissue was associated with subsequent PCa diagnosis. However, in this study, no difference was found in the Gleason score between *P. acnes* positive and negative patients [32]. Kakegawa et al. reported that patients with high serum PSA level and initial biopsy negative for cancer progressed more frequently to PCa in subsequent biopsies if the initial biopsy was positive for the presence of *P. acnes* [51]. Interestingly, the potential pathogenic role of *P. acnes* was also assessed in the genitourinary tract by Manente et al. The researchers evaluated the presence of *P. acnes* DNA in urine or seminal fluid of patients with recurrent symptoms of urinary infection. The test results in these patients for the most common urinary tract pathogens and sexually transmitted infection (STI) agents were negative. In the conducted tests, the presence of *P. acnes* was detected in 56 urine samples (108 urine samples were examined) and in 17 semen samples (51 semen samples were examined). The authors suggested that *P. acnes* infection could be a cause of pathogenic cascade leading in the long term, an inflammatory process of the prostate tissue [41]. Any microorganism can infect the prostate gland when ascending the urethra or by reflux of urine into the prostatic duct [52].

## 4. *P. acnes* May Also Contribute to Other Cancers and Inflammatory Diseases

Recent studies show that *P. acnes* is also considered a contributing factor in the development of neoplasms in tissues other than the prostate gland [53,54,55,56]. Therefore, the potential role of this bacterium in carcinogenesis seems to be so important that it attracts the interest of more and more researchers. For instance, *P. acnes* was investigated also as a non-*Helicobacter pylori (H. pylori)* bacteria that can stimulate gastric cancer (GC) risk [53,56]. Researchers used 16S rRNA gene sequencing and fluorescent in situ hybridization (FISH) and they found that *P. acnes* significantly increased in GC tissues, especially in *H. pylori*–negative samples. Moreover, it has been investigated that the abundance of the bacteria correlated with TNM stages of GC patients. The same authors detailed the mechanism for the tumor-promoting effect of *P. acnes*. They used immunofluorescence, RT-qPCR, and Western-blotting analysis to detect that *P. acnes* triggers M2 polarization of macrophages via TLR4/PI3K/Akt signaling. Ultimately, the researchers identified *P. acnes* as a possible agent that could regulate the tumor microenvironment by enhancing immunosuppression and thus promoting GC progression [53]. It has been reported that that M2 polarization of macrophages in tumors could be driven by canonical M2 stimuli, such as IL-4, IL-10, and IL-13 [57]. It has been also found that IL-10 expression at mRNA level was greatly enhanced in macrophages stimulated with *P. acnes* [53]. On the other hand, Tzeng et al. examined the microbiome of human breast tissue, including breast cancer samples. They reported that benign tissue samples (healthy control and high-risk) have a similar microbiome composition with higher mean relative abundances of 11 genera, including *Propionibacterium* spp. However, in this study, *Propionibacterium* spp. was not found in cancer-associated samples (tumor and tumor adjacent normal) [55]. Kim et al. researched microbiome markers of pancreatic cancer based on bacteria-derived extracellular vesicles acquired from blood samples. The study showed that at the genus level, four species, including *Propionibacterium*, were less abundant, while the other six species were more abundant in pancreatic cancer samples [58]. Suprewicz et al. assessed the effect of *P.acnes* on the proliferation capability and mechanical features of gingival cells and cell lines derived from breast, lung, and ovarian cancer. It was observed that *P. acnes* had the highest growth-promoting abilities in relation to breast cancer MCF-7 and ovarian cancer SKOV-3 cells [59]. These studies suggest that *P. acnes* may be crucial agent for the development of cancer diseases. However, the role of this bacterium in carcinogenesis may depend on the type of tissue in which the tumor develops.

The activity of *P. acnes* as a proinflammatory agent has also been researched in the case of multisystem inflammatory disorders such as sarcoidosis. Studies show that *P. acnes* can be involved in the pathogenesis of sarcoidosis. The bacterium has been detected in granulomas of some sarcoidosis patients, but not in any non-sarcoidosis ganulomas, such as tuberculosis and sarcoid reaction granulomas [60,61]. Beijer et al. investigated that the presence of *P. acnes* in granulomas is associated with chronic disease requiring treatment [44]. In patients with sarcoidosis, an increased immune response to *P. acnes* antigens was observed. Shupp et al. observed that BAL cells of sarcoidosis patients produce inflammatory cytokines (TNF-α and GM-CSF) upon stimulation with *P. acnes* [62]. This suggests that *P. acnes* can be an important factor causing hyperinflammatory status also in non-cancer diseases and in various organs.

## 5. *P. acnes* Induces Proinflammatory Response in Prostate Gland

*P. acnes* cause inflammatory diseases through their hemolytic, cytotoxic, and immunostimulatory activities [63,64]. It indicates that *P. acnes* may contribute to cancer development by enhancing proinflammatory responses, as well as by modifying the prostate extracellular environment. The proinflammatory response consists of the producing chemotactic factors, recruitment, and expansion of immune cells. The inflammatory infiltrate primarily includes T lymphocytes, macrophages and, less frequently, plasma cells and eosinophils [13,49,65,66]. Infiltrating cells are inducing to release pro-inflammatory cytokines, such as tumor necrosis factor-α (TNF-α), vascular endothelial growth factor (VEGF), IL-1β, IL-6, IL-8, IL-12, and IL-17 [67]. In a study by Shinohara et al., C57BL/6J mice were inoculated with a vehicle control or a prostatectomy-derived strain of *P. acnes* strain. Researchers have observed severe acute and chronic inflammation of the prostate gland. In addition, it was investigated that the inflammatory lesions were associated with an increase in the Ki-67 proliferative index, and a decrease in the production of Nkx3.1 and androgen receptor (AR). It was also reported that the observed response required live bacteria. This indicates the potential intracellular presence of *P. acnes* in prostate epithelial cells [68]. Davidsson et al. observed increased cell proliferation and cytokine/chemokine secretion in the prostate cells (PNT1A cell line) that were co-cultured with isolates of *P. acnes* [46]. Similar effects have been noted previously by Fassi Fehri et al. The microarray analysis they carried out showed a strong multifaceted inflammatory response of the prostate epithelial cell line RWPE1 that was co-cultured with live *P. acnes* isolated from cancerous prostates. The authors observed active secretion of cytokines and chemokines, such as IL-6 and IL-8 from infected cells. It has been suggested that the immune response included the activation of the COX2-prostaglandin, the plasminogen-matrix metalloproteinase pathways, as well as the activation of the transcriptional factors NF-κB and STAT3. It was also found that long-term exposure to *P. acnes* altered cell proliferation and initiated cellular transformation [48]. It should be noted that an increased level of IL-6 in the serum of patients with PCa is associated with advanced metastases. Moreover, IL-6 activates the JAK/STAT signaling pathway. Persistent activation of STAT3 transcription factor induces proliferation and tumor growth. In addition, the previously mentioned molecules such as VEGF and COX-2 are involved in angiogenesis [48,69,70,71,72].

## 6. Treg and Th17 Cells in the Tumor Environment

**Figure 2:**
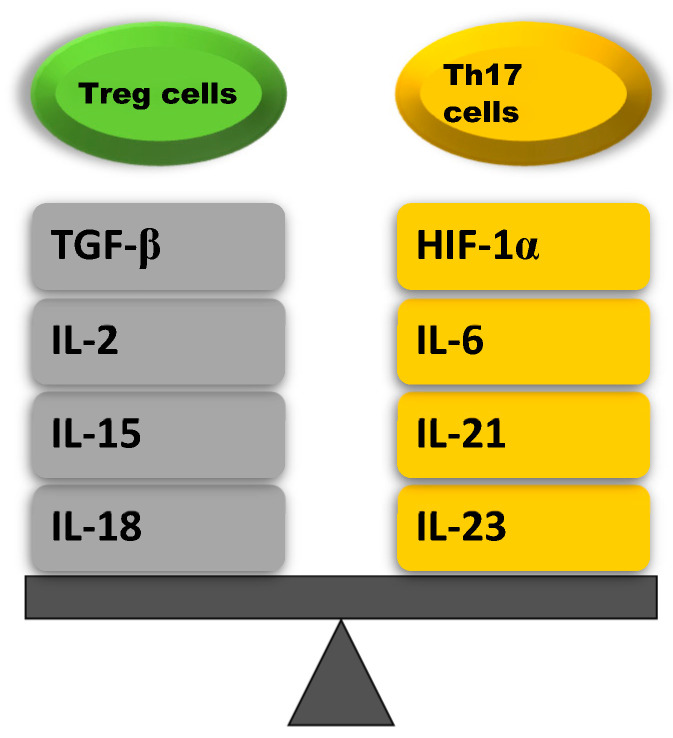
Cytokines that maintain the balance between Treg and Th17 cells [75].

Although T helper type 17 (Th17) cells and Treg cells share a common precursor cell (the naïve CD4 T cell) and require a common tumor growth factor (TGF)-β signal for initial differentiation, they have opposite functions. Th17 cells induce autoimmunity and inflammation, whereas the role of Treg cells is to inhibit these activities and maintain immune homeostasis [73]. The balance between Th17 and Treg cells is mainly affected by TCR signaling, cytokines, costimulatory signals, microbiomes, and other factors. It has been found that mainly inflammation cytokines (IL-2, IL-6, IL-15, IL-18, IL-21, and IL-23), including transforming growth factor *β* (TGF-*β*) and hypoxia-inducible factor 1-α *(HIF-1α)*, are involved in regulating the balance between Th17 and Treg cells. IL-2, IL-15, IL-18, and TGF-*β affect Treg cells, whereas IL-6, IL-21, IL-23*, and *HIF-1α affect Th17 cells* (Figure 2, Table 1) [71,74].

The process of Th17 cells differentiation consists of three stages in which various factors are involved, such as TGF-*β*, IL-6, IL-21, and IL-23. The initiation of Th17 cell differentiation is mediated by TGF-*β* and IL-6. Then, IL-21 expands the differentiation state of these cells. Ultimately, IL-23 is responsible for maintaining the stable maturation of Th17 cells during the later stage of the differentiation process [76]. Interestingly, naïve CD4^+^ T cells in the absence of IL-6 or IL-21 differentiate into Treg cells [75]. Treg cells are chemoattracted to the tumor microenvironment by chemokine gradients. CCR4, CCR8, CCR10, and CXCR3 induce Treg cell migration to the tumor microenvironment in response to CC and CXC chemokines: CCR4 is bound by CCL17 and CCL22, CCR8 is bound by CCL1, CCR10 is bound by CCL28,and CXCR3 is activated by CXCL9/10/11 [77]. Treg cells are highly activated and immunosuppressive within the tumor microenvironment. These cells are characterized by upregulated levels of FoxP3 and Helios [78,79,80]. Thus, Treg cells play an essential role in maintaining immune tolerance and balance, whereas Th17 cells show proinflammatory activities [74]. Treg cells suppress effector cells, such as T effector (Teff) cells, monocytes, macrophages, natural killer (NK) cells, and antigen-presenting (APC) cells via various mechanisms that lead to the inhibition of effector cell activation and proliferation, as well as to the induction of apoptosis [81]. These mechanisms include increased consumption of IL-2 and Teff deprivation and upregulated levels of inhibitory immune checkpoints [82,83,84,85]. Treg cells suppress the activity of immune cells and thereby controlling inflammation, by secreting anti-inflammatory cytokines, such as TGF-*β* and IL-10 [86,87,88].

Th17 are the key mediators of many autoimmune diseases; therefore, they can be also involved in the inflammatory process of cancer. [89]. As research shows, Th17 cells have been found in various human cancers. It has been also reported that Th17 cells in cancer show both tumor-promoting and tumor-suppressing activity. Th17-derived cytokines: IL-17 and IL-22, promote transformed cell properties and neighboring stromal cell activity, thereby influence the tumor microenvironment. Moreover, these cytokines also modulate the activities of myeloid and T cells that are involved in regulation of the immune system [90]. Liu et al. investigated that age-related CD4+ T cells, especially Th17 cells-secreted factors, can contribute to prostate carcinogenesis. The researchers used a C57BL/6J (B6) mouse as an ageing animal model to determine the role of age-related Th17 response in PCa cell growth, migration, and invasion. It was observed that Th17 cells, Th17 cytokines, and Th17/Treg ratio were increased compared to young mice. In addition, factors secreted from Th17 cells (IL-17A, IL-17F, and IL-22) promoted PCa cell viability, migration, and invasion, as well as activated the NF-κB and ERK1/2 signaling in PCa cells compared to young mouse prostate tissues [91]. NF-κB has also been found as a critical link between inflammation and cancer. Many researchers have demonstrated a positive association between the activation of NF-κB and PCa [92,93,94]. Thus, research confirmed that the balance between Th17 and Treg cells may play an essential role in prostate carcinogenesis. As earlier studies show, IL-17A expression increased in more than 50% of prostate cancers [95,96]. It has also been revealed that IL-17 induces prostate adenocarcinoma via MMP7-mediated epithelial-to-mesenchymal transition [97]. Other researchers investigated the importance of Th17 cells and IL-17 in a Pten-null prostate cancer mouse model. They found that SR1001 and anti-IL-17 antibody treatment increased apoptosis and reduced proliferation, angiogenesis, and inflammatory cell infiltration in Pten-null mice [98]. Cunningham et al. also assessed the interleukin-17′s role in prostate cancer. They reported that co-injection of recombinant IL-17A and mouse PCa cells enhanced metastasis to the pelvic lymph nodes [99]. Research suggests that Th17 cells and IL-17 can not only contribute to tumor progression but can also increase metastasis in patients with PCa.

## 7. *P. acnes* Contributes to Prostate Infiltration by Treg and Th17 Cells

It has been found that the presence of *P. acnes* in the prostate gland in patients with BPH and PCa is connected with the higher infiltrating of prostatic tissue by regulatory T CD4(+)FoxP3(+) cells (Treg cells). The authors also noticed that the infiltration of Treg cells is dependent on the aggressiveness of cancer in the Gleason scale in patients with *P. acnes* [49]. It has been previously investigated that the number of Treg cells is significantly increased in both tumor tissue and in the peripheral blood of patients with PCa [100]. This may indicate the effect of *P. acnes* on promoting carcinogenesis in the prostate gland. Interestingly, studies have shown that Treg cells can suppress anti-tumor responses, which is directly related with increasing risk of cancer recurrence. These cells play a role in preserving self-tolerance and inhibiting extra immune responses. Hence, Treg cells may support tumor progression as tumor-associated antigens are mainly self-antigens [100]. The studies also discovered that PCa patients with elevated levels of Treg cells within the tumor microenvironment have poor prognosis and low survival rates [100,101]. As other research shows, the imbalance between Treg cells and CD4(+)IL-17(+) cells in the tumor environment may promote inflammation and cancer progression. Moreover, it can contribute to the development of acquired resistance to immunotherapy. On the other hand, targeting Treg and Th17 cells could improve clinical outcomes [101,102]. It should be noted that the role of Th17/Treg in chronic inflammation associated with various diseases has been highlighted in many other studies, for example, in obesity, inflammatory bowel disease, autoimmune, and metabolic diseases [74,103,104,105]. 

As research shows, IL-17 exerts strong pro-inflammatory effects and is an important mediator in inflammation-associated cancer [105]. Radej et al. observed that the infiltration of CD4(+)IL-17(+) cells was significantly higher in BPH patients with *P. acnes* compared to BPH patients without the presence of this bacterium in prostate tissue. However, this correlation was not found in patients with PCa [48]. On the other hand, in earlier research carried out by Steiner et al., the authors reported that IL-17 mRNA and protein expression was increased in 79% of BPH and 58% of PCa specimens. In this study, IL-17 expression was very weak and restricted to lymphocytes in the samples of normal prostate [95]. The results obtained by Agak et al. confirm that *P. acnes* can induce immune cells to release high levels of IL-17. The authors also noted that this bacterium can modulate the CD4(+) T cell response in various ways, leading to the generation of Th17 cells [106].

## 8. Conclusions

The microbiome of the prostate can contribute to the development of prostate chronic inflammation in relation to BPH, as well as to carcinogenesis and tumor progression. The research carried out on human samples so far has provided evidence for a link between BPH or PCa and *P. acnes* using various technical approaches, such as cultivation, in situ hybridization, immunohistochemistry, and PCR-based profiling of bacterial 16S rRNA. *P. acnes* as a most prevalent microorganism in the prostate gland may be a predisposing factor for inflammation that can lead to the development of BPH and PCa. The relationship between the immune response and the presence of *P. acnes* in prostate tissue shows the participation of this bacterium in the intensification of inflammation. It appears that Treg and Th17 cells can be factors that promote prostate disease in response to *P. acnes* infection. The balance between Treg and Th17 cells in BPH and PCa patients may have important implications for clinicians and clinical researchers seeking more reliable prognostic markers and more targeted therapeutic approaches. In light of the current research, the indication of *P. acnes* as the cause of prostate chronic inflammation, and thus, also the cause of prostate diseases, seems to be justified; however, it should be thoroughly investigated and clearly confirmed.

## Figures and Tables

**Table 1:** Effect of cytokines on the Treg/Th17 cells balance [75].

Treg Cells Upregulating Cytokines	Th17 Cells Upregulating Cytokines
**TGF-β**: stimulates naïve CD4+ T cells that induce SMAD2 and SMAD3 that activate the transcription factor Foxp3	**HIF-1α**: promotes the differentation of Th17 cells by inducing ROR-γt transcription and inhibits the differentation of Treg cell in an active process aimed at degradation of the Foxp3 protein
**IL-2**: increases Foxp3 expression by phosphorylation of STAT5 which binds to the Foxp3 locus	**IL-6**: stimulates naïve CD4+ T cells to differentiate into Th17 via STAT3 phosphorylation which induces the upregulation of Th17-specific genes (ROR-γt, IL-17, IL-23)
**IL-15**: increases Foxp3 expression by activating STAT5 and inhibits Th17 cell differentation by reducing IL-17 secretion	**IL-21**: stimulates Th17 cell differentation by activating STAT3, which increases ROR-γt expression
**IL-18**: inhibits Th17 cell differentation by inhibiting MyD88- dependent IL-1R downstream signal	**IL-23**: maintains Th17 cell differentation by enhancing the transcription of Th17 specyfic cytokines such as ROR-γt
